# Critical Involvement of Calcium-Dependent Cytosolic Phospholipase A2α in Aortic Valve Interstitial Cell Calcification

**DOI:** 10.3390/ijms21176398

**Published:** 2020-09-03

**Authors:** Antonella Bonetti, Lorenzo Allegri, Federica Baldan, Magali Contin, Claudio Battistella, Giuseppe Damante, Maurizio Marchini, Fulvia Ortolani

**Affiliations:** 1 Department of Medicine, Histology and Electron Microscopy Unit, University of Udine, I-33100 Udine, Italy; antonella.bonetti@uniud.it (A.B.); magali.contin@uniud.it (M.C.); maurizio.marchini@uniud.it (M.M.); 2 Department of Medicine, Genetics Unit, University of Udine, I-33100 Udine, Italy; allegri.lorenzo@spes.uniud.it (L.A.); federica.baldan@uniud.it (F.B.); giuseppe.damante@uniud.it (G.D.); 3 Department of Medicine, Statistics Unit, University of Udine, I-33100 Udine, Italy; claudio.battistella@uniud.it

**Keywords:** cytosolic phospholipase A2α, aortic valve calcification, aortic valve interstitial cell cultures, dexamethasone, ultrastructure

## Abstract

The involvement of calcium-dependent cytosolic phospholipase A2α (cPLA2α) in aortic valve calcification is not exhaustively elucidated. Here, cPLA2α expression in aortic valve interstitial cell (AVIC) pro-calcific cultures simulating either metastatic or dystrophic calcification was estimated by qPCR, Western blotting, and counting of cPLA2α-immunoreactive cells, with parallel ultrastructural examination of AVIC calcific degeneration. These evaluations also involved pro-calcific AVIC cultures treated with cPLA2α inhibitor dexamethasone. cPLA2α over-expression resulted for both types of pro-calcific AVIC cultures. Compared to controls, enzyme content was found to increase by up to 300% and 186% in metastatic and dystrophic calcification-like cultures, respectively. Increases in mRNA amounts were also observed, although they were not as striking as those in enzyme content. Moreover, cPLA2α increases were time-dependent and strictly associated with mineralization progression. Conversely, drastically lower levels of enzyme content resulted for the pro-calcific AVIC cultures supplemented with dexamethasone. In particular, cPLA2α amounts were found to decrease by almost 88% and 48% in metastatic and dystrophic calcification-like cultures, respectively, with mRNA amounts showing a similar trend. Interestingly, these drastic decreases in cPLA2α amounts were paralleled by drastic decreases in mineralization degrees, as revealed ultrastructurally. In conclusion, cPLA2α may be regarded as a crucial co-factor contributing to AVIC mineralization in vitro, thus being an attractive potential target for designing novel therapeutic strategies aimed to counteract onset or progression of calcific aortic valve diseases.

## 1. Introduction

Calcium-dependent cytosolic phospholipase A2α (cPLA2α), also known as group IVA PLA2 or PLA2G4A, is an enzyme belonging to the superfamily of PLAs that catalyses the hydrolysis of membrane phospholipids bearing arachidonic acid at the *sn*-2 position. Constitutively expressed by many cell types, cPLA2α is activated by calcium at submicromolar concentrations and phosphorylation of specific serine residues enabling enzyme translocation to endomembranes, with subsequent arachidonic acid release and production of downstream signalling molecules including lysophospholipids and eicosanoids [1,2]. In their turn, these pro-inflammatory lipid mediators are reported to act as autocrine signals enhancing cPLA2α expression [3,4], which can be additionally up-regulated by pro-inflammatory cytokines [5,6] and bacterial lipopolysaccharide (LPS) [7,8]. Consistently, increased cPLA2α expression was observed in several inflammatory diseases affecting different organs and tissues, including brain, bronchi, bones, cartilages, and atherosclerotic artery walls [9,10,11,12,13]. In contrast with these extensive studies, poor research exists on the role of cPLA2α in calcification [14,15]. In particular, enzyme involvement in calcific aortic valve stenosis is not exhaustively elucidated [16,17], although some attention was devoted to the role of non-cytosolic PLA2s in this valvulopathy [18,19,20]. In our previous studies carried out under transmission electron microscopy, the same degenerative patterns characterizing valve mineralization after in vivo calcific induction [21,22,23,24,25] were reproduced in vitro using original pro-calcific models [25,26,27,28]. Namely, primarily cultured aortic valve interstitial cells (AVICs) were subjected to either metastatic- or severe- dystrophic-like calcification treating cells with LPS, conditioned medium (CM) from LPS-stimulated macrophages, and 3.0 mM inorganic phosphate (Pi) or 2.0 mM Pi, respectively, in accordance with the well-known Pi values referred to these two types of calcification. High (≥ 2.0 mM) Pi was found to be sufficient per se to prime AVIC mineralization, and its pro-calcific effects were dose-dependent; thus, the higher the Pi concentration, the shorter the incubation time to obtain AVIC calcification. The observed pro-calcific degenerative process consisted in a progressive endomembrane dissolution with intracellular release of an acidic lipid material, referred to as PPM (phthalocyanine-positive material) because of its strong affinity for phthalocyanine cuprolinic blue. Subsequent PPM layering at AVIC edges was found to generate PPLs (phthalocyanine-positive layers) acting as major hydroxyapatite crystal nucleators together with PPL-derived multivesicular bodies and their further end products, consisting in paracrystallin calcospherulae. Raman microspectroscopy also revealed close co-localization between calcium salt precipitates and lipid deposits in aortic valve leaflets affected by actual calcific stenosis [29,30]. Due to its phospholipid-degrading activity, we hypothesized that cPLA2α may play a direct role in priming and advancing the peculiar lipid-release-associated AVIC pro-calcific degeneration we previously described. Using in vitro pro-calcific models as above, possible involvement of cPLA2α in both metastatic and severe dystrophic-like AVIC calcification was ascertained in the present study. Whether enzyme expression may be influenced by Pi concentration was also assayed. The obtained results indicate that (i) cPLA2α is over-expressed by calcifying AVICs, (ii) its expression rate is positively related to Pi concentration, and (iii) increases in enzyme amounts are time-dependent and consistent with the advancement of cell calcification. To some extent, anti-calcific effects were also found subsequent to enzyme inhibition.

## 2. Results

### 2.1. cPLA2α Expression in Control and Pro-Calcific AVIC Cultures

**Figure 1:**
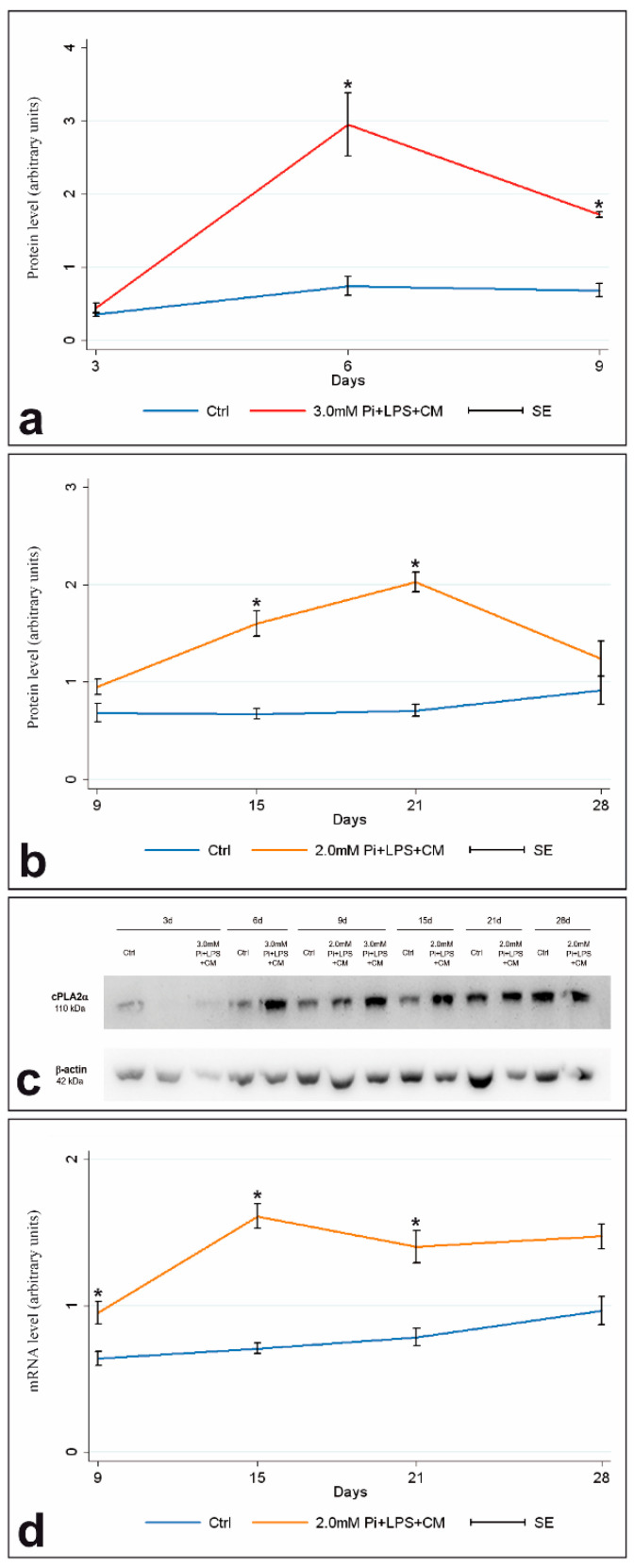
Quantification of cPLA2α in cultured AVICs. (**a**) Western blotting quantification of cPLA2α in 3- to 9-day-long control and metastatic calcification-like cultures. Protein levels are calculated as cPLA2α/β-actin, with the control being set to 1. Data are shown as mean ± SE. Statistically significant values are indicated with asterisks (*p* < 0.05). (**b**) Western blotting quantification of cPLA2α in 9- to 28-day-long control and severe dystrophic calcification-like cultures. Protein levels are calculated as cPLA2α/β-actin, with the control being set to 1. Data are shown as mean ± SE. Statistically significant values are indicated with asterisks (*p* < 0.05). (**c**) Representative Western blotting obtained from lysates of AVICs cultured as in (**a**) and (**b**). (**d**) Quantification of cPLA2α mRNA using qPCR in 9- to 28-day-long control and severe dystrophic calcification-like cultures. Data are expressed as 2^-ddCT. Data are shown as mean ± SE. Statistically significant values are indicated with asterisks (*p* < 0.05). Abbreviations: AVIC, aortic valve interstitial cell; Pi, phosphate; LPS, lipopolysaccharide; CM, conditioned medium.

**Figure 2:**
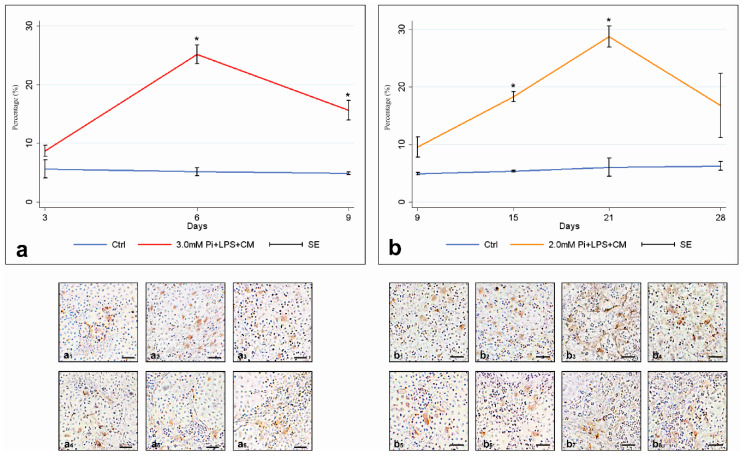
Percentages of cPLA2α-immunopositive AVICs. (**a**) Percentages of immunopositive cells in 3- to 9-day-long control and metastatic calcification-like cultures. Data are shown as mean ± SE. Statistically significant values are indicated with asterisks (*p* < 0.05). (**b**) Percentages of immunopositive cells in 9- to 28-day-long control and severe dystrophic calcification-like cultures. Data are shown as mean ± SE. Statistically significant values are indicated with asterisks (*p* < 0.05). Abbreviations: AVICs, aortic valve interstitial cells; Pi, phosphate; LPS, lipopolysaccharide; CM, conditioned medium. (**a1**–**a3**) Representative light microscopy micrographs of 3- to 9-day-long metastatic calcification-like cultures immunoreacted for cPLA2α. (**a4**–**a6**) Representative light microscopy micrographs of 3- to 9-day-long control cultures immunoreacted for cPLA2α. (**b1**–**b4**) Representative light microscopy micrographs of 9- to 28-day-long severe dystrophic calcification-like cultures immunoreacted for cPLA2α. (**b5**–**b8**) Representative light microscopy micrographs of 9- to 28-day-long control cultures immunoreacted for cPLA2α; Bar: 1 mm.

Western blotting quantification revealed cPLA2α content in 3.0-Pi-LPS-CM cultures underwent a significant time-dependent increase between days 3 and 6 (*p* < 0.025), followed by a remarkable drop at day 9, although values remained significantly higher than those in controls (Figure 1a,c). For 2.0-Pi-LPS-CM cultures (Figure 1b,c), enzyme content underwent significant time-dependent increases up to day 21 (*p* < 0.0125), with values significantly higher than those in controls starting from day 15. A remarkable drop in enzyme amounts occurred at day 28, with values resulting nearly identical to those in controls. In paralleled 2.0-Pi-LPS-CM cultures, qPCR-estimated cPLA2α mRNA amounts underwent significant time-dependent increases up to day 15 (*p* < 0.0125), with values significantly higher than those in controls starting from day 9. A moderate decrease at day 21 followed, with values remaining nearly unchanged up to day 28 (Figure 1d). For 3.0-Pi-LPS-CM cultures, qPCR analyses revealed cPLA2α mRNA content to be significantly increased compared to controls at days 6 and 9, with values showing no significant differences to each other over time (*p* > 0.025) (not shown). Compared to Western blotting estimations, a similar trend resulted for percentages of cPLA2α-immunopositive AVICs. Namely, for 3.0-Pi-LPS-CM cultures, immunopositive cells underwent a significant time-dependent increase between days 3 and 6 (*p* < 0.025), followed by a remarkable drop at day 9, with values remaining significantly higher than those in controls (Figure 2a). For 2.0-Pi-LPS-CM cultures, immunopositive AVICs underwent significant time-dependent increases up to day 21 (*p* < 0.0125), with values being significantly higher than those in controls starting from day 15. A remarkable drop occurred at day 28, with percentages of cPLA2α-positive AVICs reaching values close to those in controls (Figure 2b). Figure 2a1–a3 shows immunopositive AVICs in 3-, 6-, and 9-day-long 3.0-Pi-LPS-CM cultures. Figure 2a4–a6 shows immunopositive AVICs in 3-, 6-, and 9-day-long control cultures. Figure 2b1–b4 shows immunopositive AVICs in 9-, 15-, 21-, and 28-day-long 2.0-Pi-LPS-CM cultures. Figure 2b5–b8 shows immunopositive AVICs in 9-, 15-, 21-, and 28-day-long control cultures.

### 2.2. Effects of cPLA2α Inhibition on Pro-Calcific AVIC Cultures

**Figure 3:**
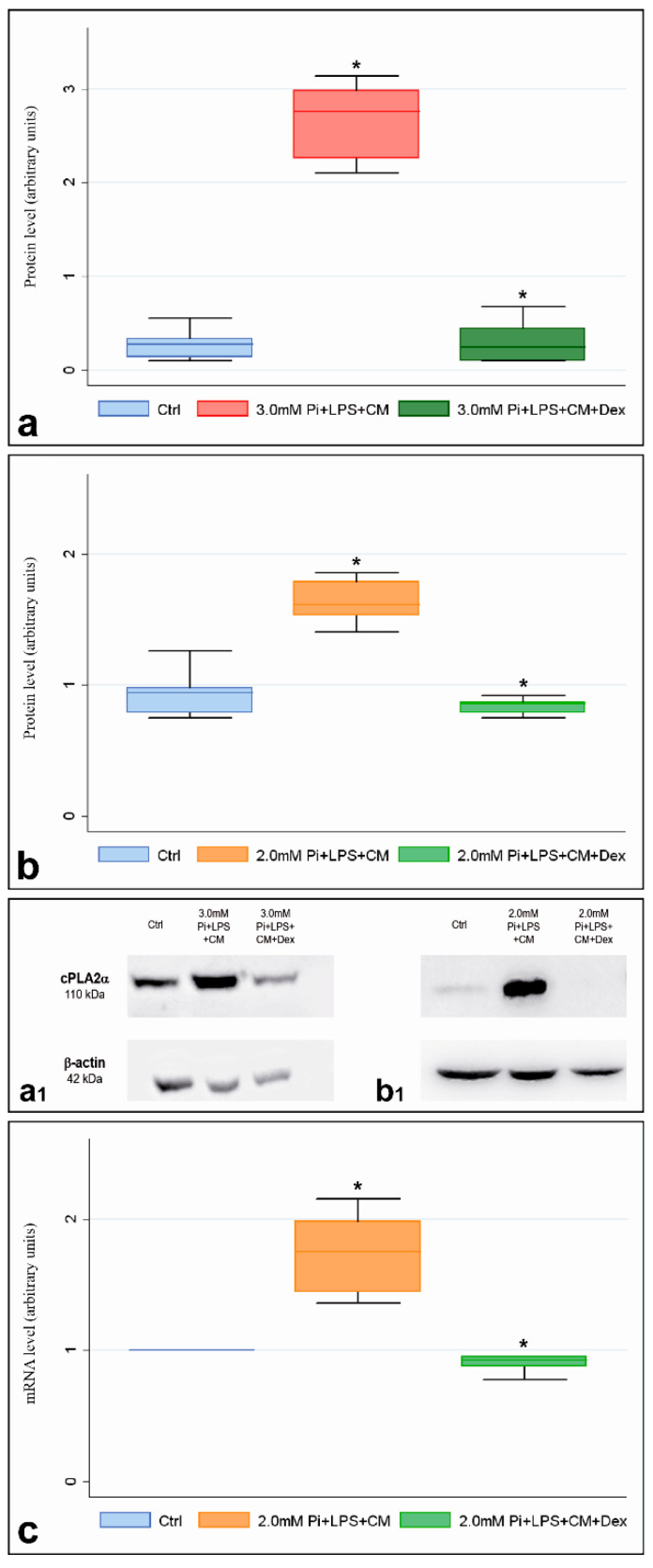
Effects of cPLA2α inhibitor dexamethasone on pro-calcific AVIC cultures. (**a**) Western blotting quantification of cPLA2α in 6-day-long control and metastatic calcification-like cultures with or without dexamethasone supplementation. Protein levels are calculated as cPLA2α/β-actin, with the control being set to 1. Data are shown as mean ± SE. Statistically significant values are indicated with asterisks (*p* < 0.05). (**b**) Western blotting quantification of cPLA2α in 21-day-long control and severe dystrophic calcification-like cultures with or without dexamethasone supplementation. Protein levels are calculated as cPLA2α/β-actin, with the control being set to 1. Data are shown as mean ± SE. Statistically significant values are indicated with asterisks (*p* < 0.05). (**a1**) Representative Western blotting obtained from lysates of AVICs cultured as in a. (**b1**) Representative Western blotting obtained from lysates of AVICs cultured as in (**b**). (**c**) Quantification of cPLA2α mRNA using qPCR in 21-day-long control and severe dystrophic calcification-like cultures with or without dexamethasone supplementation. Data are expressed as 2^-ddCT. Data are shown as mean ± SE. Statistically significant values are indicated with asterisks (*p* < 0.05). Abbreviations: AVIC, aortic valve interstitial cell; Pi, phosphate; LPS, lipopolysaccharide; CM, conditioned medium; Dex, dexamethasone.

**Figure 4:**
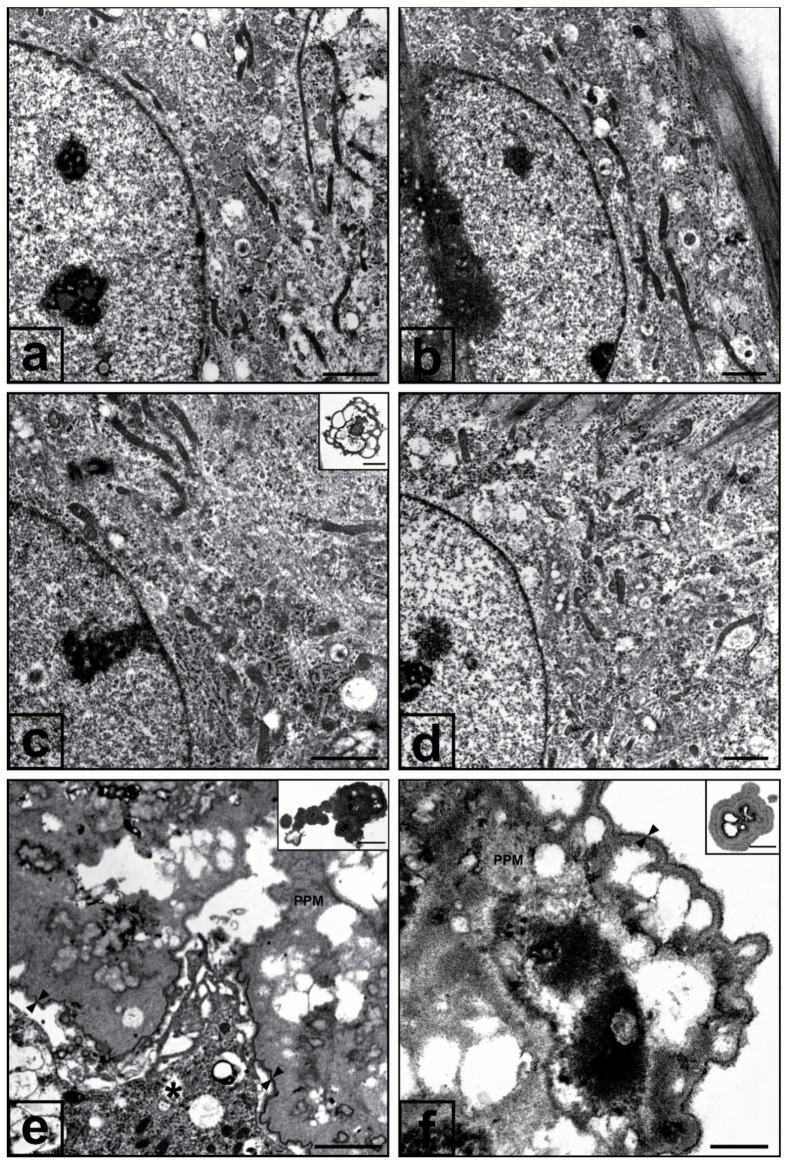
Transmission electron microscopy micrographs of cultured AVICs. (**a**) AVIC from a 6-day-long control culture showing normal features, including well preserved organelles. (**b**) AVIC from a 21-day-long control culture still showing regular organelles. (**c**) AVIC from a 6-day-long pro-calcific 3.0-Pi-LPS-CM culture supplemented with dexamethasone, showing preserved organelles as in (**a**); Inset: PPL-lined multivesicular cell remnant derived from AVIC pro-calcific degeneration as a sporadic feature. (**d**) AVIC from a 21-day-long pro-calcific 2.0-Pi-LPS-CM culture supplemented with dexamethasone, showing preserved organelles as in (**b**). (**e**) AVICs from a 6-day-long pro-calcific 3.0-Pi-LPS-CM culture showing either early degenerative features (asterisk) or advanced cell degeneration, including formation of electron-dense PPM, peripheral PPLs (counterposed arrowheads), and calcospherulae (inset). (**f**) AVIC from a 21-day-long pro-calcific 2.0-Pi-LPS-CM culture showing advanced degenerative features, including formation of intracellular PPM, peripheral PPLs (counterposed arrowheads), and cell-derived end products (inset). Bar: 0.5 μm (a), (b), (c), (d), (f); 1 μm (e), (f inset); 0.25 μm (c inset), (e inset).

Western blotting assays revealed cPLA2α amounts in 3.0-Pi-LPS-CM-Dex cultures (Figure 3a,a1) and 2.0-Pi-LPS-CM-Dex cultures (Figure 3b,b1) were significantly less than those in their counterparts not subjected to dexamethasone supplementation, with enzyme content reaching values identical to those in controls for both cultures. Occurrence of dexamethasone-dependent inhibition of gene expression was also observed, since qPCR-estimated cPLA2α mRNA content in 2.0-Pi-LPS-CM-Dex cultures was significantly less than that in 2.0-Pi-LPS-CM cultures, with values very close to those in controls (Figure 3c). An analogous inhibitory effect also resulted for cPLA2α mRNA amounts in 3.0-Pi-LPS-CM-Dex cultures (not shown). Ultrastructurally, normal features as those characterizing AVICs from 6- (Figure 4a) and 21-day-long control cultures (Figure 4b) were observed for most AVICs from 3.0-Pi-LPS-CM-Dex cultures (Figure 4c) and 2.0-Pi-LPS-CM-Dex cultures (Figure 4d). Advanced pro-calcific patterns showing the genesis of PPL-lined multivesicular cell remnants and/or calcospherulae were more frequently observed in 3.0-Pi-LPS-CM-Dex cultures (Figure 4c inset) than 2.0-Pi-LPS-CM-Dex cultures, although pro-calcific end products were far fewer than those usually detectable in 3.0-Pi-LPS-CM cultures (Figure 4e, 4e inset) and 2.0-Pi-LPS-CM cultures (Figure 4f, 4f inset), respectively. AVICs from control Dex cultures showed normal features, revealing that no dexamethasone-dependent cell alterations took place (not shown).

## 3. Discussion

In the present study, cPLA2α involvement was ascertained in AVIC calcification using pre-validated in vitro pro-calcific models, indicating the enzyme to be over-expressed by calcifying AVICs, with time-dependent enzyme increases being closely associated with progression of the inherent cell calcific degeneration. Quantitative estimations revealed cPLA2α and its mRNA to be constitutively expressed at low rates by control AVICs, undergoing significant time-dependent increases in the pro-calcific cultures exclusively. In the latter, cPLA2α over-expression can be conceivably ascribed to simultaneous cell exposition to high Pi, LPS, and CM-derived pro-inflammatory mediators. Consistently, LPS and pro-inflammatory stimuli are widely reported to act as potent cPLA2α up-regulators [5,6,7,8], whereas Pi is known to contribute to enzyme activation [2]. Further evidence that Pi could directly activate gene expression in vitro [31,32] raises the intriguing question whether direct Pi-mediated activation of cPLA2α mRNA synthesis can also take place in cultured AVICs. Although this query needs more in-depth investigation, the fact that remarkable increases in cPLA2α mRNA amounts were restricted to the pro-calcific AVIC cultures suggests that an additional pro-transcriptional effect of high Pi on the cPLA2α gene cannot be excluded. Taking also into account that higher levels of enzyme content were reached in metastatic calcification-like AVIC cultures even within shorter incubation times compared to severe dystrophic calcification-like ones, the cPLA2α expression rate appears to be positively related to Pi concentration. Evidence that high-phosphate-mediated inflammation can cause vascular cell calcification [33,34] leads to the assumption that cPLA2α over-expression by calcifying AVICs may be due to a possible pro-inflammatory effect of high phosphate, boosting that exerted by the employed pro-inflammatory stimuli. Occurrence of cPLA2α over-expression in the employed pro-calcific cultures was also consistent with failure of enzyme cleavage by caspase-3 [35,36], supporting our previous finding that apoptosis is not involved in the induced cell calcification processes [28]. Despite cPLA2α over-expression being associated with cell osteoblastic differentiation in vitro [37,38], it is worth noting that, in the present study as well as our previous ones on AVIC calcification in vitro [26,27,28], cells exhibiting osteoblast-like features were never encountered at the ultrastructural level, with the sole cell fate consisting in the described process of lipid-release-associated pro-calcific cell death. This intracellular release of pro-calcific lipid material we previously reported to occur during AVIC mineralization actually might depend on massive cell membrane dissolution subsequent to cPLA2α over-expression, since time-dependent increases in enzyme content were found to be closely associated with progression of cell mineralization. The pro-calcific role played by cPLA2α might also be exerted via the downstream activation of alkaline phosphatase, an additional enzyme contributing to mineralization progression [39]. Consistently in analogous AVIC cultures [27,28], maximum alkaline phosphatase activity was observed at incubation times longer than those corresponding to the maximal cPLA2α amounts found in the present study. This assumption is also consistent with previous reports showing cPLA2α-dependent activation of alkaline phosphatase in vitro [40,41]. Activity of downstream enzymes involved in the arachidonic acid metabolism was reported to be associated with the occurrence of calcific aortic valve stenosis [42,43], strongly suggesting that cPLA2α may actually be involved in calcific valve disease priming also in vivo. On the other hand, decreases in cPLA2α content observed at the longest incubation times may be due to pro-calcific cell degeneration, with associated enzyme breakdown. Far more substantial decreases in mRNA and enzyme amounts resulted for the pro-calcific AVIC cultures supplemented with the anti-inflammatory agent dexamethasone [44], as previously reported for other culture types [45,46]. Even taking into account the non-specific inhibitory effect of dexamethasone, the drastic lowering in mineralization as observed ultrastructurally for the pro-calcific AVIC cultures strongly suggests that AVIC calcification advances hand-in-hand with the found time-dependent cPLA2α over-expression. The identification of sporadic mineralizing cells in 3.0-Pi-LPS-CM-Dex cultures and, to a much lesser extent, 2.0-Pi-LPS-CM-Dex cultures suggests that cPLA2α can be regarded as a co-factor in AVIC calcification, high Pi being a pro-calcific factor per se, as previously reported [27,28]. High Pi is capable of activating sodium/phosphate cotransporter PiT-1 [47,48], alkaline phosphatase [49,50], and oxidative responses [51,52], also triggering cell calcification in absence of cPLA2α over-expression, although at much lower degrees. This leads to speculation that sole inflammation-lowering therapies might not be effective enough for hyperphosphatemic patients.

In conclusion, the obtained results indicate that (i) cPLA2α contributes to in vitro AVIC calcification, with its over-expression occurring in parallel with AVIC calcific progression and its inhibition resulting in drastic mineralization lowering, and (ii) cPLA2α expression rate is positively related to Pi concentration, with higher enzyme levels being reached in metastatic calcification-like cultures within shorter incubation times compared to severe dystrophic calcification-like ones.

## 4. Materials and Methods

### 4.1. AVIC Treatments

Primary cultures of bovine AVICs were obtained as previously described [26,27,28]. Aortic valves were retrieved from hearts of bovines slaughtered in a local abattoir (Salumificio Pitaccolo G. Srl, Castions di Strada, Udine, Italy) respecting the Reg CE 1099/2009, September 24, 2009, which regulates the protection of animals at the time of killing. Cows were not killed specifically for the purpose of this study, and no experiments were performed on them before slaughtering. The following AVIC cultures were prepared: (i) control cultures treating AVICs with Dulbecco’s modified eagle’s medium (DMEM; Sigma-Aldrich) plus 10% foetal bovine serum (Gibco) for 3, 6, 9, 15, 21, and 28 days; (ii) metastatic calcification-like cultures treating AVICs with a pro-calcific medium containing DMEM, plus 10% foetal bovine serum, plus 100 ng/mL LPS from *Escherichia coli* (Sigma-Aldrich), plus 1/5 (*v*/*v*) CM obtained from LPS-stimulated bovine macrophages [26,27,28], plus 3.0 mM Pi for 3, 6, and 9 days (3.0-Pi-LPS-CM cultures); and (iii) severe dystrophic calcification-like cultures treating AVICs with a pro-calcific medium as in (ii) but containing 2.0 mM Pi for 3, 9, 15, 21, and 28 days (2.0-Pi-LPS-CM cultures). For each AVIC culture, media were refreshed every three days. Concerning CM preparation, bovine blood was collected by a veterinarian during routine care in the whole respect of normal animal behaviour and wellness, according to the professional ethics of FNOVI (National Federation of the Orders of Italian Veterinarians) approved on 12 June 2011.

### 4.2. Western Blotting Quantification of cPLA2α

Triplicates of control cultures, 3.0-Pi-LPS-CM cultures, and 2.0-Pi-LPS-CM cultures were prepared (*n* = 3). After medium removal, AVICs were washed twice with cold PBS, scraped away, and collected into total lysis buffer (50 mM Tris HCl, 120 mM NaCl, 5 mM EDTA, 1% Triton X-100, 1% NP40, 1 mM DTT, pH 8). Cell lysates were electrophoresed on SDS-PAGE and then transferred to nitrocellulose membranes (GE Healthcare, Chicago, IL, USA). Membranes were incubated with 1:1000 rabbit anti-cPLA2α polyclonal antibody (Novus Biologicals; Cat.#: NBP2-19809; recombinant protein encompassing a sequence within the center region of human PLA2G4A) or 1:4000 rabbit anti-β-actin polyclonal antibody (Sigma-Aldrich; Cat.#: A2066; produced using C-terminal actin fragment, C11 peptide, as immunogen) overnight. Then, membranes were incubated with 1:6000 peroxidase-conjugated anti-rabbit antibody (Merck KGaA; Cat.#: 12-348) for 2 h. Blots were developed using UVITEC Alliance LD with the SuperSignal Technology (Thermo Scientific, Waltham, MA, USA).

### 4.3. qPCR Quantification of cPLA2α mRNA

Triplicates of control cultures, 3.0-Pi-LPS-CM cultures, and 2.0-Pi-LPS-CM cultures were prepared (*n* = 3). After medium removal, AVICs were washed twice with cold PBS, scraped away, and treated with RNeasy mini kit (Qiagen, Hilden, Germany) according to the manufacturer’s instructions for total mRNA extraction. Then, 500 ng of total mRNA was reverse transcribed to cDNA using random exaprimers and SuperScript III reverse transcriptase (Life Technologies, Carlsbad, CA, USA). Real-time PCR was performed using PowerUp Sybr Green Master Mix (Life Technologies, Carlsbad, CA, USA) on the QuantStudio3 Real Time PCR System (Applied BioSystems, Foster City, CA, USA). To calculate mRNA levels the ∆∆CT method via the SDS software (Applied Biosystems, Foster City, CA, USA) was used. β-actin mRNA levels were used as endogenous control. All oligonucleotides were purchased from Sigma-Aldrich.

### 4.4. Immunocytochemical Detection of cPLA2α-Positive AVICs

Triplicates of control cultures, 3.0-Pi-LPS-CM cultures, and 2.0-Pi-LPS-CM cultures were prepared seeding AVICs onto 24 × 24 mm cover glasses placed into 35 × 10 mm Petri dishes (*n* = 3). On expiry of each incubation time, AVICs were fixed with 3% PBS-buffered paraformaldehyde for 10 min and then treated with (i) 0.1% PBS-diluted Triton X-100 for 10 min, (ii) 3% PBS-diluted hydrogen peroxide for 5 min, (iii) 3% PBS-diluted normal serum for 40 min, (iv) 1:100 PBS-diluted rabbit anti-cPLA2α polyclonal antibody (Novus Biologicals; Cat. #: NBP2-19809; recombinant protein encompassing a sequence within the center region of human PLA2G4A) for 90 min at r.t., (v) 1:600 PBS-diluted peroxidase-conjugated anti-rabbit antibody (Jackson ImmunoResearch; Cat. #: 711-036-152) for 30 min, and (vi) DAB chromogen (BioGenex, Fremont, CA, USA) prepared according to the manufacturer’s instructions for 6 min. As endogenous control, primary antibody was replaced with normal serum. After mild counterstaining with hematoxylin, cover glasses were mounted on microscope slides using an aqueous mounting medium. Photographic recording was made using a Zeiss AxioImager photomicroscope. Percentages of immunopositive AVICs were estimated after cell count using ImageJ software (https://imagej.net/Fiji/Downloads).

### 4.5. cPLA2α Inhibition in Pro-Calcific AVIC Cultures

AVICs were cultured in (i) pro-calcific medium as for 3.0-Pi-LPS-CM cultures supplemented with 100 nM dexamethasone (Sigma-Aldrich, St. Louis, MO, USA) for 6 days (3.0-Pi-LPS-CM-Dex cultures) and (ii) pro-calcific medium for 2.0-Pi-LPS-CM cultures supplemented with 100 nM dexamethasone for 21 days (2.0-Pi-LPS-CM-Dex cultures). Treatment with dexamethasone was carried out for 6 and 21 days, since maximal cPLA2α amounts were estimated at these incubation times for 3.0-Pi-LPS-CM cultures and 2.0-Pi-LPS-CM cultures, respectively. To ascertain whether actual cPLA2α inhibition occurred, parallel 6-day-long 3.0-Pi-LPS-CM cultures and 21-day-long 2.0-Pi-LPS-CM cultures, including their respective control cultures, were prepared. According to the manufacturer’s instructions, dexamethasone was dissolved in absolute ethanol and additionally diluted in DMEM. After dexamethasone supplementation, the final ethanol concentration in the pro-calcific media was equal to 0.0002%. However, to ascertain whether or not toxic effects occurred on AVICs, 6- and 21-day-long control cultures supplemented with 100 nM dexamethasone were also prepared (control-Dex cultures). All AVIC treatments were performed in triplicate (*n* = 3). For each treatment, AVIC cultures were subjected to Western blotting and qPCR, as above, as well as transmission electron microscopy.

### 4.6. Transmission Electron Microscopy

Cells were fixed with a 25 mM sodium acetate/acetic acid buffer containing 2.5% glutaraldehyde, 0.05% cuprolinic blue (Electron Microscopy Sciences, Hatfield, PA, USA), and 0.05 M magnesium chloride overnight at room temperature, keeping Petri dishes on a rocking platform. Cells were then post-fixed with 2% osmium tetraoxide (Agar Scientific, Stansted, Essex, UK), dehydrated with graded ethanol solutions, and embedded into Epon 812 resin. Ultrathin sections were collected onto formvar-coated 2 × 1 mm slot copper grids and contrasted with uranyl acetate and lead citrate. Observations and photographic recording were made using a Philips CM12 STEM transmission electron microscope.

### 4.7. Statistical Analysis

Continuous variables were summarized as mean ± standard error. Data obtained from qPCR, Western blotting, and immunocytochemical estimations were tested for normal distribution using the Shapiro–Wilk test. Variation of data between groups (controls and pro-calcific cultures) throughout incubation times was explored using the analysis of variance (ANOVA) for repeated measures. Paired comparisons between groups (controls and pro-calcific cultures) for each incubation time were performed using the Student’s *t*-test or Mann–Whitney test, as appropriate. Comparisons between incubation times within each group (controls and pro-calcific cultures) were performed using the paired Student’s *t*-test or Wilcoxon signed rank test. Bonferroni correction for multiple comparisons was applied.

## 5. Conclusions

The present results strongly suggest that cPLA2α over-expression by calcifying AVICs is crucial in triggering and sustaining their dramatic lipid-release-associated pro-calcific degeneration in vitro. Hence, cPLA2α could be seen as an attractive target in conceiving novel therapeutic strategies aimed to prevent or mitigate actual calcific aortic valve disorders.

## Abbreviations

cPLA2α	Calcium-dependent cytosolic Phospholipase A2α
AVICs	Aortic Valve Interstitial Cells
LPS	Lipopolysaccharide
PPM	Phthalocyanine-Positive Material
PPL	Phthalocyanine-Positive Layer
Pi	Inorganic Phosphate
CM	Conditioned Medium
Dex	Dexamethasone
